# Subdural hematoma expansion in relation to measured mean and peak systolic blood pressure: A retrospective analysis

**DOI:** 10.3389/fneur.2022.1026471

**Published:** 2022-10-17

**Authors:** Keegan Plowman, David Lindner, Edison Valle-Giler, Alex Ashkin, Jessica Bass, Carl Ruthman

**Affiliations:** ^1^Graduate Medical Education Internal Medicine Residency, NCH Healthcare System, Naples, FL, United States; ^2^Division of Pulmonary Critical Care Medicine, NCH Healthcare System, Naples, FL, United States; ^3^Division of Neurological Surgery, NCH Healthcare System, Naples, FL, United States

**Keywords:** subdural hematoma (SDH), systolic blood pressure (SBP), hematoma expansion (HE), hemorrhage, critical care

## Abstract

**Objective:**

Subdural hematomas (SDH) account for an estimated 5 to 25% of intracranial hemorrhages. Acute SDH occur secondary to rupture of the bridging veins leading to blood collecting within the dural space. Risk factors associated with SDH expansion are well documented, however, there are no established guidelines regarding blood pressure goals in the management of acute SDH. This study aims to retrospectively evaluate if uncontrolled blood pressure within the first 24 h of hospitalization in patients with acute SDH is linked to hematoma expansion as determined by serial CT imaging.

**Methods:**

A single center, retrospective study looked at 1,083 patients with acute SDH, predominantly above age 65. Of these, 469 patients met the inclusion criteria. Blood pressure was measured during the first 24 h of admission along with PT, INR, platelets, blood alcohol level, anticoagulation use and antiplatelet use. Follow-up CT performed within the first 24 h was compared to the initial CT to determine the presence of hematoma expansion. Mean systolic blood pressure (SBP), peak SBP, discharge disposition, length of stay and in hospital mortality were evaluated.

**Results:**

We found that patients with mean SBP <140 in the first 24 h of admission had a lower rate of hematoma expansion than those with SBP > 140. Patients with peak SBP > 200 had an increased frequency of hematoma expansion with the largest effect seen in patients with SBP > 220. Other risk factors did not contribute to hematoma expansion.

**Conclusions:**

These results suggest that blood pressure is an important factor to consider when treating patients with SDH with medical management. Blood pressure management should be considered in addition to serial neurological exams, repeat radiological imaging, seizure prophylaxis and reversal of anticoagulation.

## Introduction

Subdural hematomas (SDH) are a common subtype of intracranial hemorrhage occurring in an estimated 5 to 25% of patients after head trauma (1). Known risk factors associated with SDH expansion include age, gender, alcohol use, anticoagulation, antiplatelet medications, thrombocytopenia, prolonged partial thromboplastin time (PTT) and prolonged international normalized ratio (INR) (2–6). Expanding SDH can lead to increased intracranial pressure (ICP) putting patients at risk for midline shift and brain stem herniation leading to increased morbidity and mortality.

SDH that do not meet criteria for early surgical intervention are managed conservatively with close monitoring, often in the ICU, with serial neurological exams, repeat radiological imaging, seizure prophylaxis and reversal of anticoagulation (2). Blood pressure management is physician-dependent, as blood pressure goals in patients with SDH is not yet well-described in the literature. The INTERACT trial showed that reduction of systolic blood pressure (SBP) in patients with intracerebral hemorrhage led to decreased hematoma expansion (7) and the American Heart Association (AHA) and American Stroke Association (ASA) recommend that blood pressure should be lowered in patients with SBP >220 mmHg. They suggest it is safe to aggressively lower blood pressure to an SBP of ~140 mmHg in patients with SBP ranging from 150 to 220 mmHg (8). It is unknown whether these parameters for blood pressure control for intracerebral hemorrhage are applicable to SDH as the pathophysiology greatly differs. To date there is insufficient evidence to support the practice. This study aims to retrospectively evaluate whether uncontrolled blood pressure within the first 24 h of hospitalization in patients with acute SDH is linked to hematoma expansion as determined by serial CT imaging.

## Methods

This is a single-center, retrospective observational study. Institutional review board (IRB) approval was obtained from the Naples Community Hospital (NCH) IRB. No written consent was required as this was a retrospective study. Electronic medical record data were collected for clinical purposes and no additional tests or interviews were necessary. All data were de-identified and stored in HIPAA-secured environments. With the help of the IT department, a list of the patients over the age of 18 who were admitted to NCH Healthcare System through the emergency department between 01/01/2015 and 12/31/2021 was created using the SDH ICD diagnosis codes S06.5X9A (Subdural hematoma, acute), S06.5X0A (Subdural hematoma following injury), I62.00 (Subdural hematoma, non-traumatic) and I62.03 (Subdural hematoma, chronic). This resulted in a list of 1,083, predominantly above age 65, patients. Manual chart review was then conducted on each patient. Patients were excluded if they did not receive an initial head CT in the emergency department with follow up head CT no later than 24 h after admission, or if SDH was not clearly visualized on initial CT. Patients were also excluded with chronic SDH without an acute component, chronic subdural hygromas, post-operative SDH, concurrent large intraparenchymal or subarachnoid hemorrhage, or those who underwent neurosurgical intervention prior to repeat head CT.

Additional background data collected included age at time of admission, sex, race, ethnicity, admission date and time, discharge date and time and discharge disposition. Patient charts were then manually reviewed to collect initial CT head date and time, repeat CT head date and time, hemoglobin, hematocrit, platelets, blood urea nitrogen (BUN), creatinine, prothrombin time (PT), INR, PTT, blood alcohol level and use of anticoagulation or antiplatelet agents.

The primary author was responsible for data oversight and maintaining the integrity of the data. Quality control was performed throughout data collection and statistical analysis. The data that support the findings of this study are available upon reasonable request from the corresponding author.

### Blood pressure measurement

All SBP measurements over the first 24 h, starting from patient arrival at the ED, were collected. Mean SBP was calculated for each patient and patients were divided into three groups: mean SBP <140, 140–159, and >160 mmHg. The peak SBP value was also recorded for each patient, and they were divided into six groups: < 140, 140–159, 160–179, 180–199, 200–219, and >220 mmHg.

### Hematoma expansion

Hematoma expansion was defined as >2 mm increase in size, positive leakage sign, defined as >10 percent Hounsfield unit increase in density of subdural hematoma on CT scan (9), new midline shift or requiring surgical intervention for a change in status within 24 h of admission. A 2 mm increase in size was chosen to avoid radiologist bias in subdural hematoma measurements. Hematoma expansion was recorded as a yes or no. SDH thickness measurement was based on the slice with largest area and hematoma density was measured in Hounsfield Units.

#### Primary endpoint

The primary endpoint was hematoma expansion in relation to both mean and peak SBP.

#### Secondary endpoint(s)

Secondary endpoints include hospital length of stay, disposition and in hospital mortality in relation to mean SBP, peak SBP and hematoma expansion.

### Statistical analysis

Patients were divided into their respective groups based on mean and peak SBP values. Chi-square analysis was then performed on each group to evaluate its relationship with frequency of hematoma expansion.

Logistic regression was performed with hematoma expansion as the dependent variable and mean SBP and peak SBP as the predictive factors. Age, sex, anticoagulation, antiplatelet therapies, active alcohol use, thrombocytopenia, prolonged PTT, and prolonged INR were added as factors in addition to both the mean SBP analysis and the peak SBP. There were too few cases of thrombocytopenia to test for an interaction effect of thrombocytopenia and mean or peak SBP.

The secondary endpoint of length of stay was evaluated using one-way ANOVA. The secondary endpoint of discharge disposition was evaluated using chi-square analysis. Patients discharged to inpatient psychiatric facility or transferred to long term care facility were excluded due to insufficient numbers for analysis. There were not enough data points to determine statistical differences when evaluating the secondary endpoint of in-hospital mortality.

## Results

Of the initial 1,083, 614 patients were excluded leaving a total of 469 patients included in the study, of which, 108 patients (23%) met criteria for hematoma expansion. The criteria for hematoma expansion can be seen in Table 1.

**Table 1:** Hematoma expansion criteria and size of subdural hematoma on repeat CT head.

	**Total 108 (100%)**
**Hematoma expansion criteria**, ***n*** **(%)**
2 mm increase in size	55 (51%)
>10% increase in hounsfield units	6 (5.5%)
New midline shift	47 (43.5%)
**Size of subdural hematoma** ***n*** **(%)**
<5 mm	3 (3%)
5 to 10 mm	59 (54.5%)
>10 mm	46 (42.5%)

### Baseline characteristics

The baseline characteristics including age, sex, race, ethnicity, admission labs and time between CT scans are reported in Table 2. There were complete admission labs for 420 of the 469 patients as detailed in Table 2.

**Table 2:** Baseline characteristics of patients.

**Age, mean** **±SD (range)**	75.89 ± 14.903 (19–101)
<65 years old, ***n*** (%)	82 (17.3%)
>65 years old, ***n*** (%)	388 (82.7%)
**Sex**
Female, ***n*** (%)	216 (46.1%)
Male, ***n*** (%)	253 (53.9%)
**Race**, ***n*** **(%)**
Asian	4 (0.9%)
African American	4 (0.9%)
White	440 (93.8%)
Other	18 (3.8%)
Unknown	3 (0.6%)
**Ethnicity**, ***n*** **(%)**
Hispanic or latino	36 (7.7%)
Not hispanic or latino	429 (91.9%)
Unknown	2 (0.4%)
**Admission labs, mean** **±SD (range)**
Hemoglobin	12.88 ± 2.033 (6.0–17.8)
Hematocrit	39.39 ± 16.681 (15.4–53.9)
Platelets	211.97 ± 77.43 (9.0–571.0)
Blood Urea Nitrogen (BUN)	19.84 ± 10.045 (4.0–91.0)
Creatinine	1.108 ± 0.706 (0.4–8.9)
Prothrombin Time (PT)	14.469 ± 6.46 (9.4–88.5)
International Normalized Ratio (INR)	1.196 ± 0.620 (0.8–7.4)
Partial Thromboplastin Time (PTT)	18.055± 6.353 (19.0–84.6)
Time between CT scans, mean ± SD (range)	12:55 ± 6:07 (1:10–28:58)

#### Risk factors for hematoma expansion

In this study, 388 (83%) patients were older than 65 years of age which is consistent with the aging population at our hospital system. There was a distribution of 253 (53.9%) men and 216 (46.1%) women. There were 82 (17.5%) patients on anticoagulation, 153 (32.6%) patients using antiplatelet medication and 11 (2.3%) patients with thrombocytopenia, as defined as platelets >50,000, at time of diagnosis of SDH. A positive blood alcohol level was found in 52 (11.1%) patients at time of admission. We observed 29 (6.2%) patients with prolonged PTT and 80 (17.1%) patients with prolonged INR. The complete data for risk factors for hematoma expansion can be seen in Table 3.

**Table 3:** Risk factors for hematoma expansion.

	**Hematoma expansion (*n* = 108)**	**No hematoma expansion (*n* = 361)**	**Total (*n* = 469)**
**Age**, ***n*** **(%)**			
<65	15 (13.9%)	66 (18.3%)	81
>65	93 (86.1%)	295 (81.7%)	388
**Sex**, ***n*** **(%)**			
Male	64 (59.3%)	189 (52.4%)	253
Female	44 (40.7%)	172 (47.6%)	216
**Anticoagulation**, ***n*** **(%)**			
Apixaban	8 (7.4%)	17 (4.7%)	25
Warfarin	11 (10.2%)	29 (8.0%)	40
Dabigatran	0 (0.0%)	5 (1.4%)	5
Rivaroxaban	1 (0.9%)	11 (3.0%)	12
Not taking	88 (81.5%)	299 (82.8%)	387
**Antiplatelet therapies**, ***n*** **(%)**			
Aspirin	39 (36.1%)	97 (26.9%)	136
Clopidogrel	5 (4.6%)	11 (3.0%)	16
Prasugrel	0 (0.0%)	1 (0.3%)	1
Not taking	64 (59.2%)	252 (69.8%)	316
**Active alcohol use**, ***n*** **(%)**
Yes	8 (7.4%)	44 (12.2%)	52
No	16 (14.8%)	36 (10.0%)	52
Not tested	84 (77.8%)	281 (77.8%)	365
**Thrombocytopenia** **<** **50**, ***n*** **(%)**			
Yes	5 (4.6%)	6 (1.7%)	11
No	103 (95.4%)	353 (97.8%)	456
Not tested	0 (0.0%)	2 (0.6%)	2
**Prolonged PTT**, ***n*** **(%)**			
≤ 35.4	95 (88.0%)	298 (82.5%)	393
>35.4	6 (5.6%)	23 (6.4%)	29
Not tested	7 (6.5%)	40 (11.1%)	47
**INR**, ***n*** **(%)**
<1.2	88 (81.5%)	285 (78.9%)	373
>1.2	20 (18.5%)	60 (16.6%)	80
Not tested	0 (0.0%)	16 (4.4%)	16

^*^There was one patient on aspirin, clopidogrel and apixaban.

^**^There were 11 patients on aspirin + apixaban or coumadin or rivaroxaban.

^***^There were two patients on clopidogrel + coumadin or rivaroxaban.

^****^There were 27 patients on aspirin + clopidogrel.

### Primary endpoint

#### Mean SBP

**Figure 1:**
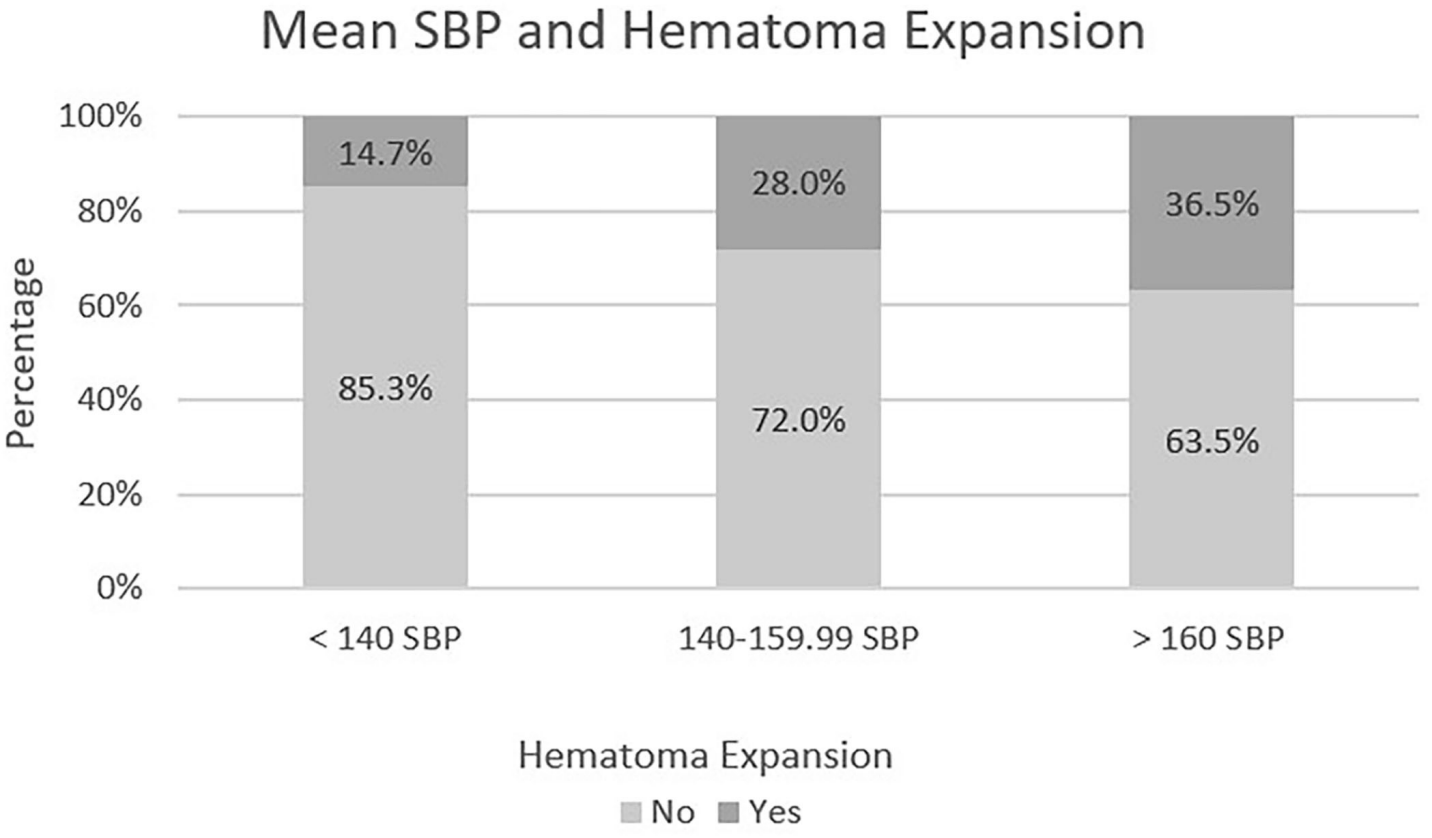
Mean SBP in relation to hematoma expansion.

The proportion of those with hematoma expansion was significantly less for the SBP Mean group of <140mmHg when compared to the SBP mean group of 140–159 mmHg [χ^2^(1) = 10.788, *p*-value < 0.01] and as compared to the SBP mean group of 160 mmHg or more [χ^2^(1) = 14.648, *p*-value < 0.01]. There was not a significant difference between the 140–159.99 mmHg mean SBP group and the mean SBP group of 160 mmHg or more. The distribution of cases can be seen in Figure 1.

Logistic regression was performed with hematoma expansion as the dependent variable and mean SBP as the predictive factor. The proportion of those with hematoma expansion was significantly less for the mean SBP group <140 mmHg when compared to the mean SBP group of 160 mmHg or more [Waldχ^2^(1) = 13.725, *p*-value < 0.01]. Mean SBP group of 160 mmHg or more was 3.324 times more likely to have hematoma expansion than mean SBP group <140 mmHg. There was no significant difference between mean SBP group 140–159.99 mmHg when compared to mean SBP group of 160 mmHg or more. The following variables were not significant when added as a factor in addition to mean SBP: age, sex, anticoagulation, antiplatelet therapies, active alcohol use, thrombocytopenia, prolonged PTT and prolonged INR; each with *p*-values > 0.05. The *p*-value was 0.059 for aspirin when antiplatelet therapies was included with the mean SBP group as independent variables. The *p*-value for the significance of thrombocytopenia within the regression model was 0.051. There were too few cases of thrombocytopenia to test for an interaction effect of thrombocytopenia and mean SBP group on hematoma expansion.

#### Peak SBP

**Figure 2:**
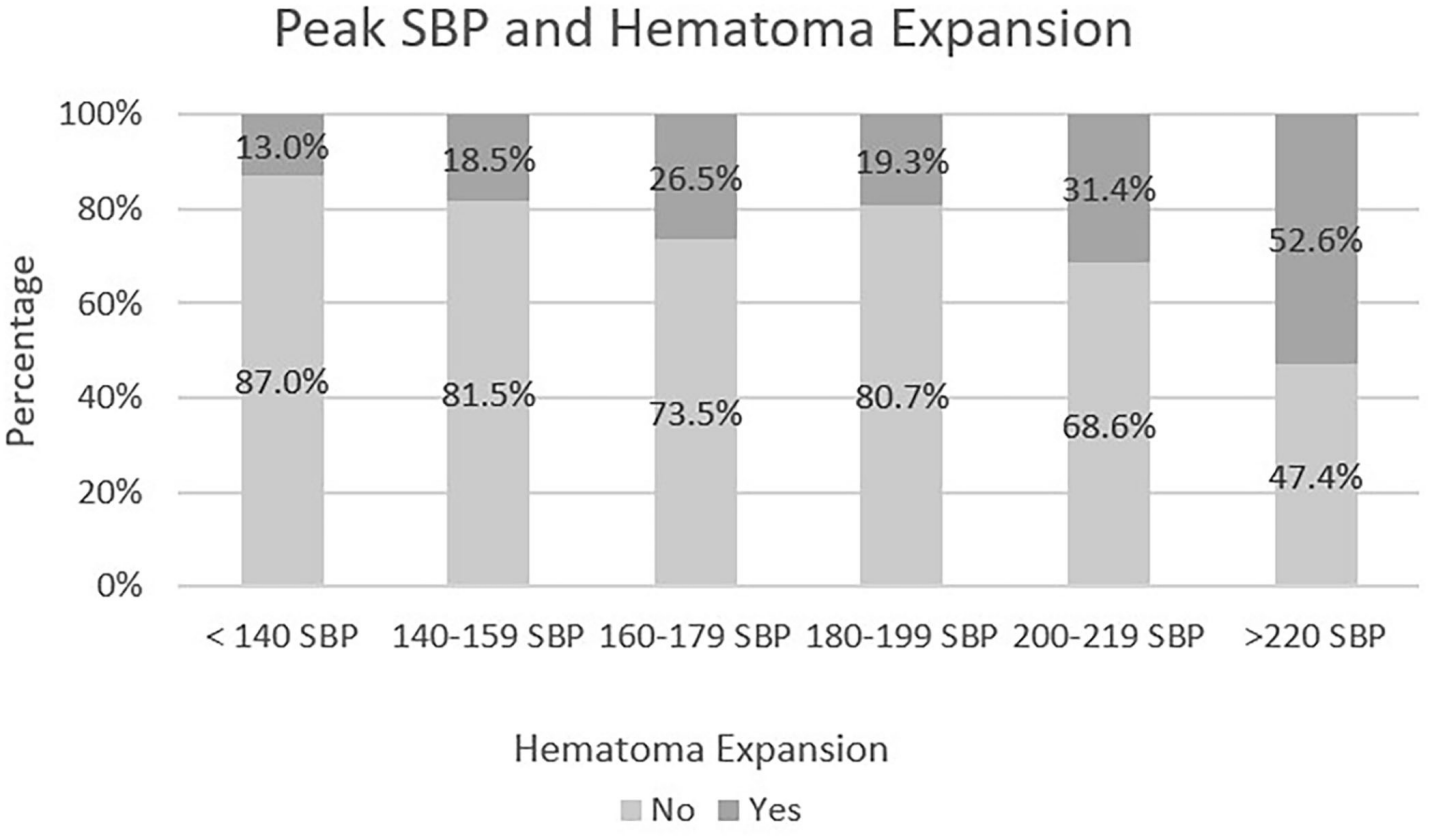
Peak SBP in relation to hematoma expansion.

Overall, there was a significant difference in the proportion of those with hematoma expansion between groups [χ^2^(1) = 15.079, *p*-value < 0.05]. The proportion of those with hematoma expansion was significantly greater for the peak SBP group >220 mmHg when compared to each of the other five groups, each at the 5% level of significance. The only other pairwise comparison that showed a significant difference in the proportion of hematoma expansion was between the peak SBP group <140 mmHg and peak SBP group 200–219 mmHg, [χ^2^(1) = 4.051, *p*-value < 0.05]. The distribution of cases can be seen in Figure 2.

**Figure 3:**
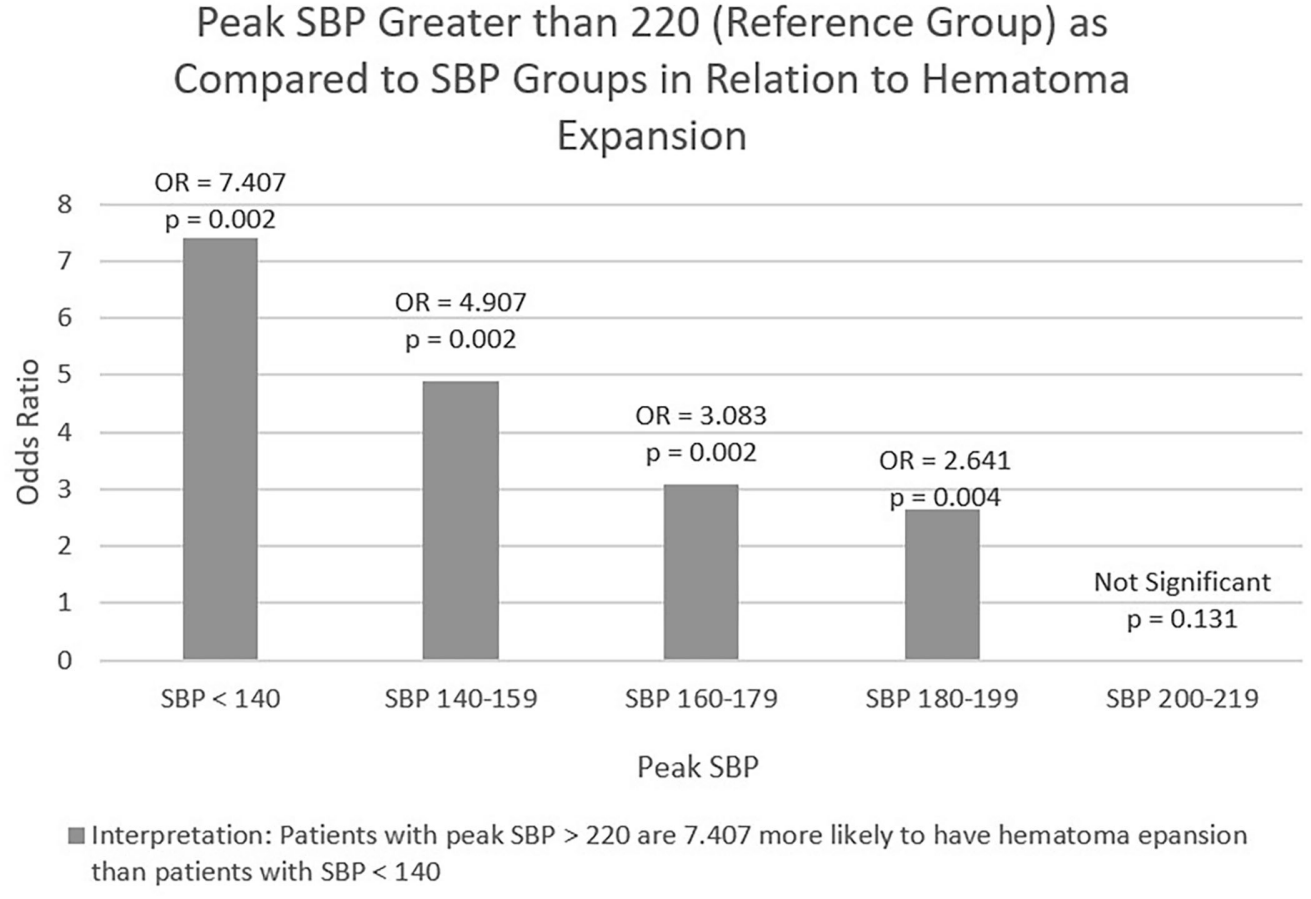
Logistic regression of peak SBP and hematoma expansion.

Logistic regression was performed with hematoma expansion as the dependent variable and peak SBP as the predictive factor. The following figure gives the significance for each Peak SBP group as well as the odds ratio for each group in comparison to the reference group (SBP > 220 mmHg). As detailed in Figure 3, the SBP group >220 mmHg was 7.407 times more likely to have hematoma expansion when compared to SBP Group <140 mmHg. There was not a statistically significant relationship between SBP > 220 mmHg and SBP Group 200–219, *p* = 0.131. As mentioned above there was a statistically significant relationship between peak SBP and SBP group <140 mmHg [χ^2^(1) = 4.051, *p*-value < 0.05]. A single reading of SBP > 220 mmHg was associated with an increased frequency of hematoma expansion.

The following variables were not significant when added as a factor in addition to peak SBP: age, sex, anticoagulation, antiplatelet therapies, active alcohol use, thrombocytopenia, prolonged PTT, and prolonged INR; each with *p*-values > 0.05. The *p*-value for the main effect significance of thrombocytopenia within the regression model was 0.034, but there were too few cases of thrombocytopenia to test for an interaction effect of thrombocytopenia and peak SBP group on hematoma expansion.

### Secondary endpoint

#### Length of stay

There was no significant difference in mean length of stay between mean SBP groups, using one-way ANOVA [*F*(2, 466) = 1.234, *p*-value > 0.05]. One-way ANOVA revealed a significantly longer mean length of stay in those with hematoma expansion, [*F*(1, 467) = 11.048, *p*-value < 0.01].

#### Discharge disposition

There were no significant differences between mean SBP group and discharge disposition [χ^2^(6) = 8.052, *p*-value > 0.05]. Overall, the proportion of those with hematoma expansion differs significantly between discharge dispositions [χ^2^(3) = 14.868, *p*-value < 0.01]. Pairwise comparisons revealed significant differences in the proportion of those with hematoma expansion between home and hospice [χ^2^(1) = 4.613, *p*-value < 0.05] and between home and transfer to skilled nursing facility (SNF) [χ^2^(1) = 12.819, *p*-value < 0.01]. This is detailed below in Table 4.

**Table 4:** Discharge disposition in relation to hematoma expansion.

	**Hematoma expansion (*n* = 108)**	**No hematoma expansion (*n* = 361)**	**Total (*n* = 469)**
Length of stay in days, mean ± SD	6.40 ± 7.755	4.33 ± 4.890	
**Disposition**, ***n*** **(%)**
Home	28 (25.9%)	159 (44.0%)	187
Home with home healthcare	18 (16.7%)	79 (21.9%)	97
Transfer to SNF	42 (38.9%)	90 (24.9%)	132
Transfer to inpatient psychiatric facility	1 (0.9%)	2 (0.6%)	3
Transfer to long term care	1 (0.9%)	0 (0.0%)	1
Left against medical advice	1 (0.9%)	3 (0.8%)	4
Hospice	10 (9.3%)	23 (6.4%)	33
Expired	7 (6.5%)	5 (1.4%)	12

#### In hospital mortality

There were insufficient numbers to determine any statistical differences in relationship to mean SBP and hematoma expansion.

## Discussion

This study showed that patients with mean SBP <140 mmHg in the first 24 h of admission had a lower rate of hematoma expansion than those with SBP > 140 mmHg. Patients with peak SBP > 200 mmHg had an increased frequency of hematoma expansion with the largest effect seen in patients with SBP > 220 mmHg. This effect was seen in patients with even a single peak SBP reading > 200. Other risk factors did not contribute to hematoma expansion when logistic regression was performed. To date, only one study has shown a positive association between SBP and change in SDH volume as calculated by the ABC/2 method (10). There are no established guidelines for blood pressure management in patients with SDH and the only guidance for optimal management has been extrapolated from intracerebral hemorrhage data which is limited due to differences in pathophysiology. Current intracerebral hemorrhage guidelines recommend reduction of blood pressure in patients with SBP > 220 (8). Early intensive reduction of SBP within the first 24 h has been shown to decrease hematoma expansion in patients with intracerebral hemorrhage with the greatest treatment effect seen in patients with the lowest SBP (7). Our results showed that uncontrolled blood pressure within the first 24 h of admission led to an increased frequency of hematoma expansion in patients with SDH.

Risk factors that have been associated with SDH expansion in the literature include age, gender, alcohol use, anticoagulation, antiplatelet medications, thrombocytopenia, prolonged PTT, and prolonged INR (2–6). In a study of traumatic brain injury, researchers found that acute SDH was the most common diagnosis seen, was most common in patients older than 65 and that male patients accounted for 56% of all cases (11). History of alcohol use is associated with an increased risk of subdural hematoma expansion (12). We observed a larger number of SDH in patients older than age 65 and in male patients but did not see an increased frequency of hematoma expansion in patients with positive blood alcohol levels. Administration of anticoagulation and anti-platelet agents as well as thrombocytopenia are known risk factors for generalized bleeding. However, the literature remains controversial and the role these factors play in increased risk for SDH expansion is unclear. The use of oral anticoagulation and antiplatelet agents has been linked to increased morbidity and mortality (13–16) but this has not been consistently reported (12). Our study found no relationship between use of anticoagulation, antiplatelet medications, thrombocytopenia, prolonged PTT or prolonged INR and increased frequency of hematoma expansion. In this patient group, hematoma expansion was associated with worse patient outcomes, further making the case for investigation into ways to lower risk factors. These patients had significantly longer length of stay and were more likely to be discharged to a skilled nursing facility or hospice rather than be discharged home.

The reason uncontrolled SBP was correlated with an increased risk of hematoma expansion is unclear, as acute SDH primarily occur secondary to rupture of the bridging veins leading to blood collecting within the dural space (2). However, it is estimated that up to 30% of SDH may be secondary to an arterial source due to rupture of cortical arteries (5). Recently embolization of the middle meningeal artery (MMA) has proven to be a viable treatment option for refractory or chronic SDH (17, 18). It is thought that fragile neovascularization of the leaky inner dura mater membrane supplied by the middle meningeal artery contributes to the development of chronic SDH (19). This is the result of a pro-inflammatory cascade that causes leaky capillary endothelial connections and angiogenesis in the setting of acute SDH (20). It is unclear if this contributes substantially to hematoma expansion in patients with acute SDH. There are numerous case reports linking traumatic aneurysm development and aneurysmal rupture of the MMA to development of acute SDH (21, 22). More evidence is needed to determine if elevated mean and peak SBP, and by extension elevated pressure within the MMA or other cortical arteries, in the first 24 h of acute SDH treatment contributes to an increase in capillary leakage or increased risk for aneurysmal rupture in a manner substantial enough to affect SDH severity.

One limitation of this study is the retrospective design. As a result of this some of the data is incomplete such as alcohol level and laboratory studies on admission. We were also unable to calculate a modified rankin score on discharge. It was not possible to examine all desired variables such as the effect size may have on SDH and relationship of SDH to trauma. This was performed at a single center and as a result may have a lack of diversity. There was not a standardized protocol for repeat radiological imaging and there was variability in the timing of the repeat scans. There is the potential for interrater variability when reading the CT scans despite criteria for hematoma expansion being designed to attempt to minimize radiologist bias. There was also variability in the number of blood pressure measurements that were taken in that not all patients had blood pressure measurements every hour. Some patients had significantly more blood pressure measurements throughout their stay than others. Only acute SDH were included in the study but the relationship in time to an inciting event was not able to be evaluated. Although data was collected over a 5-year span there is no long term follow up data on the patients. Despite the large sample size there were not enough patients within certain subgroups such as the thrombocytopenia group to perform complete statistical analysis.

The current study provides a foundation on which to design a prospective or randomized control trial. Future designs should examine risk factors such as age, gender, alcohol use, anticoagulation, antiplatelet therapies, thrombocytopenia, prolonged PTT and prolonged INR in relationship to SDH and hematoma expansion. The effect of size of hematoma and incidence of traumatic events should be examined. Efforts should be made to objectively quantify hematoma expansion using a formula such as the ABC/2 formula. Long term follow up of acute SDH will determine whether people with uncontrolled SBP had higher progression to chronic SDH and higher morbidity and mortality.

## Conclusions

These results suggest that blood pressure is an important, potentially modifiable, factor which should be addressed when treating patients with SDH with medical management. A goal of mean SBP < 140 mmHg in the first 24 h of management may decrease the risk of hematoma expansion and elevated blood pressure, particularly those greater than a systolic of 200 mmHg, should be avoided. Blood pressure management has the potential to reduce morbidity, mortality, and healthcare burden. A randomized, controlled trial with longitudinal follow-up is needed to determine optimal blood pressure goals to decrease the risk of hematoma expansion.

## Data availability statement

The raw data supporting the conclusions of this article will be made available by the authors, without undue reservation.

## Ethics statement

The studies involving human participants were reviewed and approved by NCH Institutional Review Board. Written informed consent for participation was not required for this study in accordance with the national legislation and the institutional requirements.

## Author contributions

KP designed and conceptualized study, major role in the acquisition of data, data collection and processing, interpreted and analyzed the data, and drafted and revised the article. DL designed and conceptualized study, interpreted and analyzed the data, and revised the article. EV-G revised the article. AA major role in the acquisition of data and drafted and revised the article. JB designed and conceptualized study, interpreted and analyzed the data, and drafted and revised the article. CR designed and conceptualized study and revised the article. All authors contributed to the article and approved the submitted version.

## Funding

This work was performed as part of employment under NCH Healthcare System. The authors KP, DL, EV-G, AA, JB, and CR are all employed within this system.

## Conflict of interest

The authors declare that the research was conducted in the absence of any commercial or financial relationships that could be construed as a potential conflict of interest.

## Publisher's note

All claims expressed in this article are solely those of the authors and do not necessarily represent those of their affiliated organizations, or those of the publisher, the editors and the reviewers. Any product that may be evaluated in this article, or claim that may be made by its manufacturer, is not guaranteed or endorsed by the publisher.

## Abbreviations

AHA: American Heart Association
ASA: American Stroke Association
BUN: Blood urea nitrogen
ICP: Intracranial pressure
INR: International normalized ratio
IRB: Institutional review board
NCH: Naples Community Hospital
PT: Prothrombin time
PTT: Partial thromboplastin time
SBP: Systolic blood pressure
SDH: Subdural hematoma.
