# Supplementary material for: Subdural hematoma expansion in relation to measured mean and peak systolic blood pressure: A retrospective analysis
Source: Front Neurol. 2022 Oct 17;13:1026471. doi: 10.3389/fneur.2022.1026471 (PMC9618657; doi:10.3389/fneur.2022.1026471)
Supplement: Supplementary file 1 [file Data_Sheet_1.docx]

Supplemental Table 1. Mean SBP and relation to hematoma expansion

| **SBP Mean Group** | **No Hematoma Expansion** | **Yes Hematoma Expansion** | **Total** |
| --- | --- | --- | --- |
| < 140 N | 185 | 32 | 217 |
| Percentage | 85.3% | 14.7% | 100.0% |
| 140-159.99 N | 136 | 53 | 189 |
| Percentage | 72.0% | 28.0% | 100.0% |
| > 160 N | 40 | 23 | 63 |
| Percentage | 63.5% | 36.5% | 100.0% |
| Total N | 361 | 108 | 469 |
| Percentage | 77.0% | 23.0% | 100.0% |

Supplemental Table 2. Peak SBP and relation to hematoma expansion

| **SBP Mean Group** | **No Hematoma Expansion** | **Yes Hematoma Expansion** | **Total** |
| --- | --- | --- | --- |
| < 140 N | 40 | 6 | 46 |
| Percentage | 87.0% | 13.0% | 100.0% |
| 140-159 N | 106 | 24 | 130 |
| Percentage | 81.5% | 18.5% | 100.0% |
| 160-179 N | 111 | 40 | 151 |
| Percentage | 73.5% | 26.5% | 100.0% |
| 180-199 N | 71 | 17 | 88 |
| Percentage | 80.7% | 19.3% | 100.0% |
| 200-220 N | 24 | 11 | 35 |
| Percentage | 68.6% | 31.4% | 100.0% |
| > 220 N | 9 | 10 | 19 |
| Percentage | 47.4% | 52.6% | 100.0% |
| Total N | 361 | 108 | 469 |
| Percentage | 77.0% | 23.0% | 100.0% |

Supplemental Table 3. Logistic regression of peak SBP and hematoma expansion.

| SBP Group | N | Wald Chi-Square (df = 1) | Significance | Odds Ratio |
| --- | --- | --- | --- | --- |
| < 140 | 46 | 9.956 | .002 | 7.407 |
| 140 – 159 | 130 | 9.650 | .002 | 4.907 |
| 160 – 179 | 151 | 5.173 | .023 | 3.083 |
| 180 – 199 | 88 | 8.294 | .004 | 2.641 |
| 200 – 219 | 35 | 2.282 | .131 (not significant) | NA |
| $\geq220$ | 19 | Reference Group |  |  |
| Interpretation: Patients with SBP > 220 are 7.407 more likely to have hematoma expansion than patients with SBP < 140 | | | | |

Supplemental Table 4. Mean SBP and relation to length of stay

| **Mean SBP Group** | N | Mean Length of Stay | SD for Length of Stay |
| --- | --- | --- | --- |
| < 140 | 217 | 4.37 | 3.967 |
| 140 – 159.99 | 189 | 5.26 | 7.398 |
| > 160 | 63 | 4.95 | 5.125 |
| Total | 469 | 4.81 | 5.736 |

Supplemental Table 5. Hematoma Expansion and relation to length of stay

| **Hematoma Expansion** | N | Mean Length of Stay | SD for Length of Stay |
| --- | --- | --- | --- |
| No | 361 | 4.33 | 4.890 |
| Yes | 108 | 6.40 | 7.755 |
| Total | 469 | 4.81 | 5.736 |

Supplemental Table 6. Discharge disposition in relation to mean SBP.

|  | **SBP < 140** | **SBP 140 – 159.99** | **SBP > 160** | **Total**  **(n=469)** |
| --- | --- | --- | --- | --- |
| **Length of Stay in Days, mean + SD** | 4.37 + 3.967 | 5.26 + 7.398 | 4.95 + 5.125 |  |
| **Disposition, n (%)** |  |  |  |  |
| Home | 95 (50.8%) | 71 (38.0%) | 21 (11.2%) | 187 (100.0%) |
| Home with home healthcare | 40 (41.2%) | 41 (42.3%) | 16 (16.5%) | 97 (100.0%) |
| Transfer to SNF | 56 (42.4%) | 54 (40.9%) | 22 (16.7%) | 132 (100.0%) |
| Transfer to Psych Facility | 2 (66.7%) | 1 (33.3%) | 0 (0.0%) | 3 (100.0%) |
| Transfer to Long Term Care | 0 (0.0%) | 1 (100.0%) | 0 (0.0%) | 1 (100.0%) |
| Left Against Medical Advice | 1 (25.0%) | 3 (75.0%) | 0 (0.0%) | 4 (100.0%) |
| Hospice | 19 (57.6%) | 13 (39.4%) | 1 (3.0%) | 33 (100.0%) |
| Expired | 4 (33.3%) | 5 (41.7%) | 3 (0.25%) | 12 (100.0%) |

Supplemental Table 7. Mean SBP in relation to in hospital mortality.

| **Mean SBP Group** | N | Percentage |
| --- | --- | --- |
| < 140 | 4 | 33.3% |
| 140 – 159.99 | 5 | 41.7% |
| > 160 | 3 | 25.0% |
| Total | 12 | 100.0% |

Supplemental Table 8. Hematoma expansion in relation to in hospital mortality.

| **Hematoma Expansion** | N | Percentage |
| --- | --- | --- |
| No | 5 | 41.7% |
| Yes | 7 | 58.3% |
| Total | 12 | 100.0% |
